# Visualising an invisible symbiosis

**DOI:** 10.1002/ppp3.10180

**Published:** 2021-02-11

**Authors:** Jennifer McGaley, Uta Paszkowski

**Affiliations:** ^1^ Department of Plant Sciences University of Cambridge Cambridge UK

**Keywords:** arbuscular mycorrhizal symbiosis, art, education, fractal, microscopy, public engagement

## Abstract

Despite the vast abundance and global importance of plant and microbial species, the large majority go unnoticed and unappreciated by humans, contributing to pressing issues including the neglect of study and research of these organisms, the lack of interest and support for their protection and conservation, low microbial and botanical literacy in society, and a growing disconnect between people and nature. The invisibility of many of these organisms is a key factor in their oversight by society, but also points to a solution: sharing the wealth of visual data produced during scientific research with a broader audience. Here, we discuss how the invisible can be visualised for a public audience, and the benefits it can bring.

**Summary:**

Whether too small, slow or concealed, the majority of species on Earth go unseen by humans. One such rather unobservable group of organisms are the arbuscular mycorrhizal (AM) fungi, who form beneficial symbioses with plants. AM symbiosis is ubiquitous and vitally important globally in ecosystem functioning, but partly as a consequence of its invisibility, it receives disproportionally little attention and appreciation. Yet AM fungi, and other unseen organisms, need not remain overlooked: from decades of scientific research there exists a goldmine of visual data, which if shared effectively we believe can alleviate the issues of low awareness. Here, we use examples from our experience of public engagement with AM symbiosis as well as evidence from the literature to outline the diverse ways in which invisible organisms can be visualised for a broad audience. We highlight outcomes and knock‐on consequences of this visualisation, ranging from improved human mental health to environmental protection, making the case for researchers to share their images more widely for the benefit of plants (and fungi and other overlooked organisms), people and planet.

## Graphical abstract

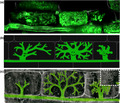

## 1 INTRODUCTION: THE ISSUES OF INVISIBILITY

Most of life on Earth is invisible to its human inhabitants. This includes the estimated one trillion microbial species that are too small to be resolved by the human visual system (Klotz, 2010; Locey & Lennon, 2016; Mora et al., 2011), the further thousands of macroscopic species that inhabit concealed habitats such as subterranean or underwater (Appeltans et al., 2012; Giller, 1996), and then the perfectly visible species that are simply overlooked, as described by the phenomenon of “plant blindness” (Sanders, 2019; Wandersee & Schussler, 2001) or “fungal blindness” (Talbot, 2020). So much of nature's diversity being invisible, literally or metaphorically, can contribute to a lack of awareness of the existence of these species, understanding about their lives, and appreciation of their importance, the knock‐on consequences of which have been well documented in the areas of education (Byrne, 2011; Knapp, 2019; Wandersee & Schussler, 2001), health (Nai et al., 2016; Timmis et al., 2019), research (“Fungus focus”, 2018; Klee, 2018; Meyer et al., 2016; Stagg et al., 2009), conservation (Balding & Williams, 2016; Macdonald et al., 2015) and the environment (Amprazis & Papadopoulou, 2020; Willis, 2018).

**FIGURE 1:**
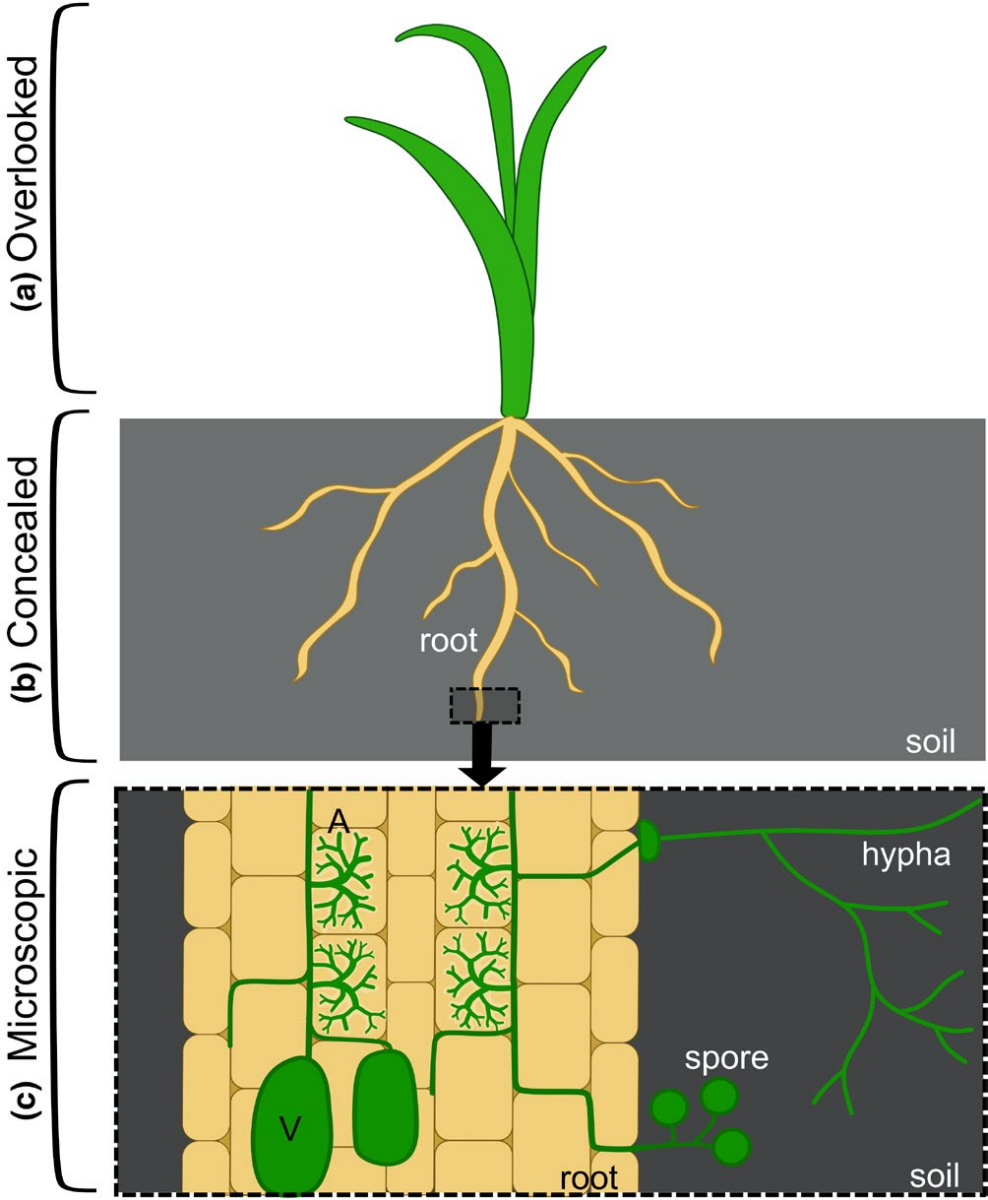
Schematic of the different levels of invisibility affecting arbuscular mycorrhizal (AM) symbiosis. (a) The above ground portions of the plant‐partner of AM symbiosis are macroscopic and visible, but often overlooked due to a bias in human perception, often termed as “plant blindness.” (b) AM fungi and the plant roots that they colonise are found underground, concealing them from human view. (c) AM symbiosis involves fungal hyphae entering the plant root, proliferating between the plant root cells and then entering plant cells and branching extensively to form nutrient exchange structures called arbuscules (A). Later in the symbiosis, fungal storage bodies (vesicles, V), and reproductive structures (spores) are formed. The symbiosis is not only concealed within the root, but the fungal structures are also microscopic

One group of organisms who exemplify the issues of invisibility are arbuscular mycorrhizal (AM) fungi. These are a group of filamentous fungi that enter into mutually beneficial symbioses with around 80% of land plant species (Tedersoo et al., 2020), colonising the plant roots and developing extensively branched intracellular structures called arbuscules, where nutrients are exchanged with their plant partner (Figure 1). AM fungi occur in all climates and soils across the globe, are critical to plant evolution, ecosystem functioning and global nutrient cycles, and have potential applications in sustainable agriculture (Chen et al., 2018; Sawers et al., 2008; Thirkell et al., 2017; Willis et al., 2013).

**FIGURE 2:**
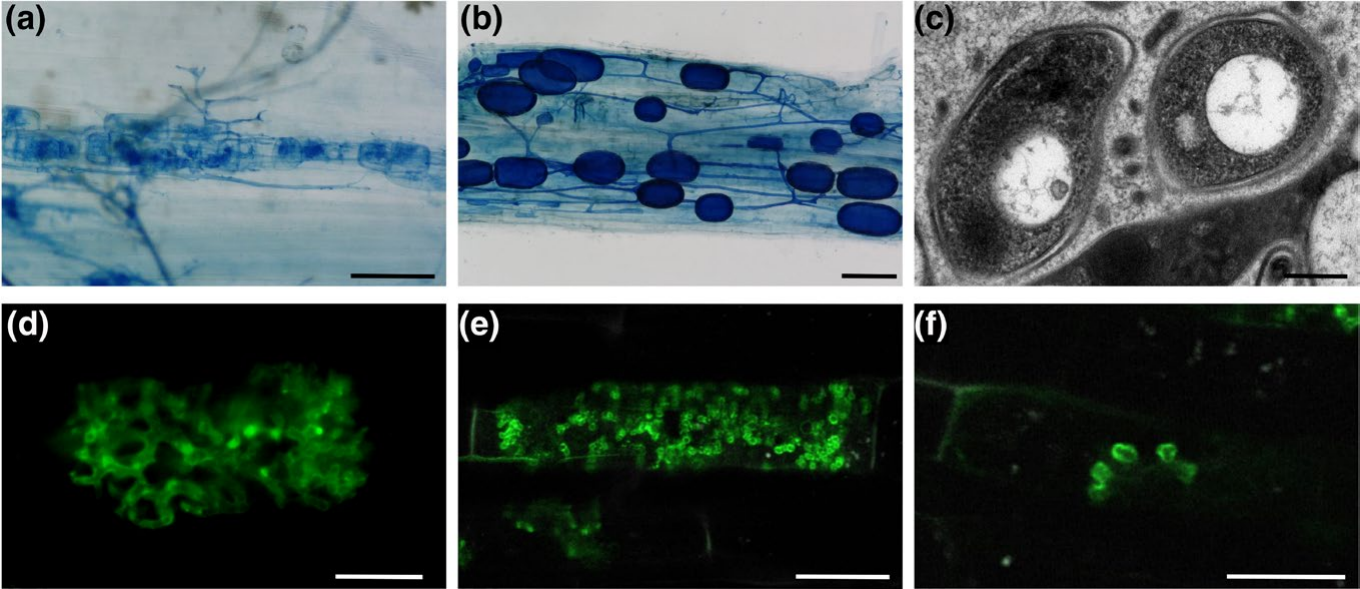
Examples of micrographs produced by a range of techniques in the study of arbuscular mycorrhizal symbiosis. (a) Brightfield micrograph of trypan‐blue stained root showing fungal hyphae and arbuscules (dark blue). Scale bar = 100 μM. (b) Brightfield micrograph of trypan‐blue stained root showing fungal hyphae and storage vesicles (dark blue). Scale bar = 100 μM. (c) Transmission electron micrograph of rice root cell colonised by AM fungus, showing cross sections of arbuscule branches. Image courtesy of Ronelle Roth. Scale bar = 200 nM. (d) Confocal scanning laser micrograph of fungal arbuscule stained with WGA‐Alexafluor^488^ (green) in cleared root tissue. Scale bar = 10 μM. (e) Confocal scanning laser micrograph of a symbiosis‐specific rice phosphate transporter tagged with GFP (green) surrounding arbuscule branches in live root tissue. Scale bar = 20 μM. (f) Multiphoton micrograph of a symbiosis‐specific rice phosphate transporter tagged with GFP (green) outlining the branches of a newly developing arbuscule. Scale bar = 20 μM

**FIGURE 3:**
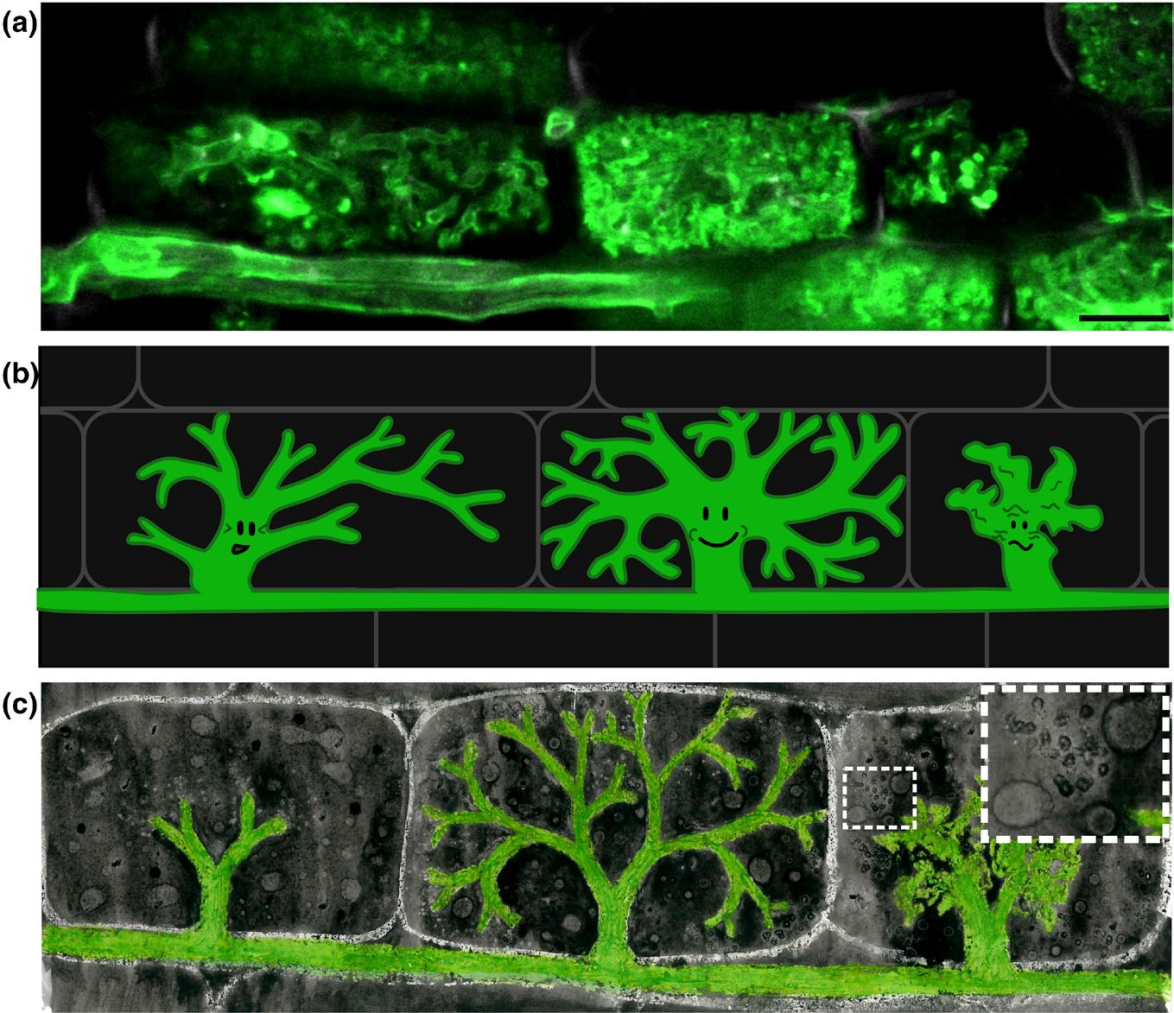
Arbuscules in a rice root visualised by: (a) confocal laser scanning microscopy of WGA‐Alexafluor^488^ –stained fungi (green). Scale bar = 10 μM. (b) Cartoon arbuscule characters. (c) Art (oil pastel and watercolour). To represent the nutrients being exchanged at the arbuscule during arbuscular mycorrhizal symbiosis, oil (lipids) and salt (minerals) were applied to the painting, giving the final texture (enlarged in image inset)

The levels of invisibility are stacked against AM fungi. First, AM symbiosis involves an interaction between plants and fungi: two kingdoms that regularly go overlooked and neglected in daily life (Knapp, 2019; Plantlife, 2008; Figure 1a). Second, AM fungi are soil‐dwelling, with their spores and hyphae found abundantly (with reports of over 100 m of hyphae and 30 spores per cubic centimetre of soil [Miller et al., 1995; Silva‐Flores et al., 2019]) but unobservable underground (Figure 1b). Third, to complete their life‐cycle, these fungi must enter into plant roots, even into plant cells, concealing them yet further. Finally, all of these fungal structures are microscopic, with most hyphae measuring under 10 μM in diameter, and arbuscules extending less than 100 μM in length (Figures 1c, 2 and 3a). This is in contrast with other fungi, including many ectomycorrhizal species, where at least a macroscopic fruiting body often marks the fungus's existence. The multiple layers of invisibility contribute to minute awareness, interest and recognition afforded to AM symbiosis, creating a mismatch between public perception (Bulunuz et al., 2008; Byrne, 2011) and ecological and societal importance of AM fungi (“Fungus focus”, 2018).

## 2 THE OPPORTUNITY: A GOLDMINE OF VISUAL DATA

Yet there is a group within society to whom AM fungi, and other naturally invisible organisms, are highly visible: the researchers who study them. As part of regular biological investigation, visual data in the form of photographs, micrographs, models and illustrations and are produced prolifically (Meijering et al., 2016). And the imaging approaches used are expanding, revealing the formerly unseen in ever increasing temporal and spatial resolution (Komis et al., 2018; Swedlow, 2012).

In the case of AM symbiosis, visualisations have existed for over 100 years, initially in the form of illustrations on the back of microscopic observations of sectioned and stained tissue (Bonfante, 2018) followed by micrographs of stained fungal structures within cleared plant roots (Phillips & Hayman, 1970; Figure 2a,b), electron micrographs of fungal and plant cell ultrastructure (Bonfante‐Fasolo, 1984; Koide & Mosse, 2004; Walker & Powell, 1979; Figure 2c) and now a rapidly increasing range of techniques, such as confocal laser scanning microscopy of stained AM fungi (Hans et al., 2004; Montero et al., 2019; Zhang et al., 2010; Figures 2d and 3a), confocal and multiphoton microscopy of fluorescent fusion proteins for live, subcellular protein localisation in arbusculated cells (Roth et al., 2018; Figure 2e,f), video microscopy of quantum dot‐tagged nutrients within AM fungal hyphae (Whiteside et al., 2019) and epifluorescence imaging of arbuscules throughout development and collapse in live roots (Kobae & Hata, 2010).

This wealth of visual data represents an opportunity to address the issues of invisibility. The images produced during research provide ample and diverse raw material for visualising the invisible for a broader audience. Here, we draw on evidence from the literature and our own experiences of public engagement with AM symbiosis to outline *how* these visuals can be shared to increase awareness, understanding, and appreciation of invisible organisms. We then discuss the potential benefits, making the case for scientists to share their visuals beyond their fields of research and academia.

## 3 THE SOLUTION: HOW TO SHOW OFF THE INVISIBLE

### 3.1 Research images

The first and simplest approach to visualising the unseen for a public audience is the sharing of raw research outputs. This can include displaying printed photos or micrographs at public engagement events, such as galleries of stained mycorrhizal fungi shown alongside the host plants (Luginbuehl & Choi, 2017), or sharing digital versions on social media platforms (e.g. Paszkowski Group, 2020).

Raw images can effectively attract attention (Balm, 2014). Humans are an incredibly visual species and are known to be drawn to images (Thorp, 2019). This is amplified by the striking and eye‐catching nature of many outputs of biological imaging, such as fluorescence micrographs with bright colours and high contrast (Ivanov & Harrison, 2018; Kokkoris et al., 2020; Figure 2d–f) electron micrographs with complex and intriguing patterns (Albornoz et al., 2020; Mareš et al., 2019; Roth et al., 2019; Figure 2c) and photographs with stunning detail (Ellis et al., 2014; Runions et al., 2017). It is a common observation from our outreach activities that passers‐bye stop in their tracks in‐front of confocal micrographs of arbuscules (e.g. Figure 3a).

Such images can also be used to effectively capture attention and interest by exploiting the unusual subject matter, harnessing people's inherent fascination with the unfamiliar, and strangeness of nature (Benko, 2020). The very fact that overlooked organisms are not seen in daily life makes them unusual and intriguing to human observers. This can be aided by the other‐worldly appearance of many organisms when magnified under the microscope or viewed in high resolution, from the strange structures of AM symbiosis (Smith & Smith, 1997), to curious rhizosphere communities (Hassani et al., 2018) to bizarre plant leaf trichomes (Dai et al., 2010), and more. This can not only help draw initial attention to invisible organisms, but also stimulates an interest and curiosity as people want to know what the unusual images are depicting. Where the organisms involved are in fact perfectly visible, but just overlooked, exposing people to less familiar views of them, such as high magnification or positioning them centre‐stage in an image, can capture an interest like a familiar view does not (Thorogood, 2020). The unfamiliar and surprising nature of many biological imaging outputs may also increase the memorability of the subject, as it is known that people remember the unexpected more effectively than the predicted (Foster & Keane, 2019).

Raw images can also be used to facilitate the formation of a strong mental model of the subject. By showing people what an organisms *really* looks like, in the form of a clear photo or micrograph (e.g. Figure 2d), it provides an image that can be stored in their mind, with which further information can be associated and remembered (Byrne, 2011; Pearson et al., 2008). We have experienced this first hand, with members of the public sharing how, after being exposed to visuals of AM fungi, they now notice and engage with information about AM symbiosis, whether in the news, books or social media. Having a clear image of AM fungi to draw on can also alter people's perception of plants and soil: where plants previously were considered a “backdrop to life,” now people report not only noticing them, but picturing the underground antics of mycorrhizal fungi, filling their roots and connecting up aboveground apparently independent individuals.

### 3.2 Adding illustration

Raw images can be complemented with illustrations or diagrams, especially where completely unfamiliar organisms or structures are involved and some orientation is required, or where direct photographic/micrographic representations are not possible. In the case of AM symbiosis, we regularly accompany micrographs with corresponding diagrams as well as cartoons (Figure 3a,b).

The addition of diagrammatic illustrations can aid understanding about the subject. Images hold the power to transfer information with no requirement for prior knowledge of language or terminology: all of the information required is in the image (SeppÄNen & VÄLiverronen, 2003; Whitehouse et al., 2006). For example, the concept and terminology associated with a mycorrhizal fungus intracellularly inhabiting a plant root can be impenetrable. While an image of a plant root containing stained fungi goes some way to showing this, a neighbouring or integrated diagram displaying a plant with its roots harbouring a correspondingly coloured fungus, which in turn extends out into the illustrated soil can give context to the micrograph and enforce the concept of two distinct organisms being involved (Figure 1).

More creative illustrations can be used to attract the attention and engagement of a broader audience, in particular younger viewers. It is known that children's attention and learning can be benefited by the use of cartoons—they can be visually attractive and also fun, which is a key element of learning in younger children (Elton‐Chalcraft & Mills, 2015). And overlooked organisms often provide fantastic raw material for such illustrations, due to their strange and characterful nature (Morel et al., 2019; Scavone et al., 2019). For instance, we exploit the quirky and alien‐like properties of branched arbuscules to create comic characters, which have amused children and adults alike at public engagement events (Figure 3b). The generation of different characters can be used to exaggerate key concepts, in the case of AMF, the living together of distinct organisms, the appearance of fungi (beyond a mould or a mushroom), and that fungi can be “good” characters, combatting the conceptions of disease and germs (Bulunuz et al., 2008; Morel et al., 2019).

### 3.3 An artistic approach

**FIGURE 4:**
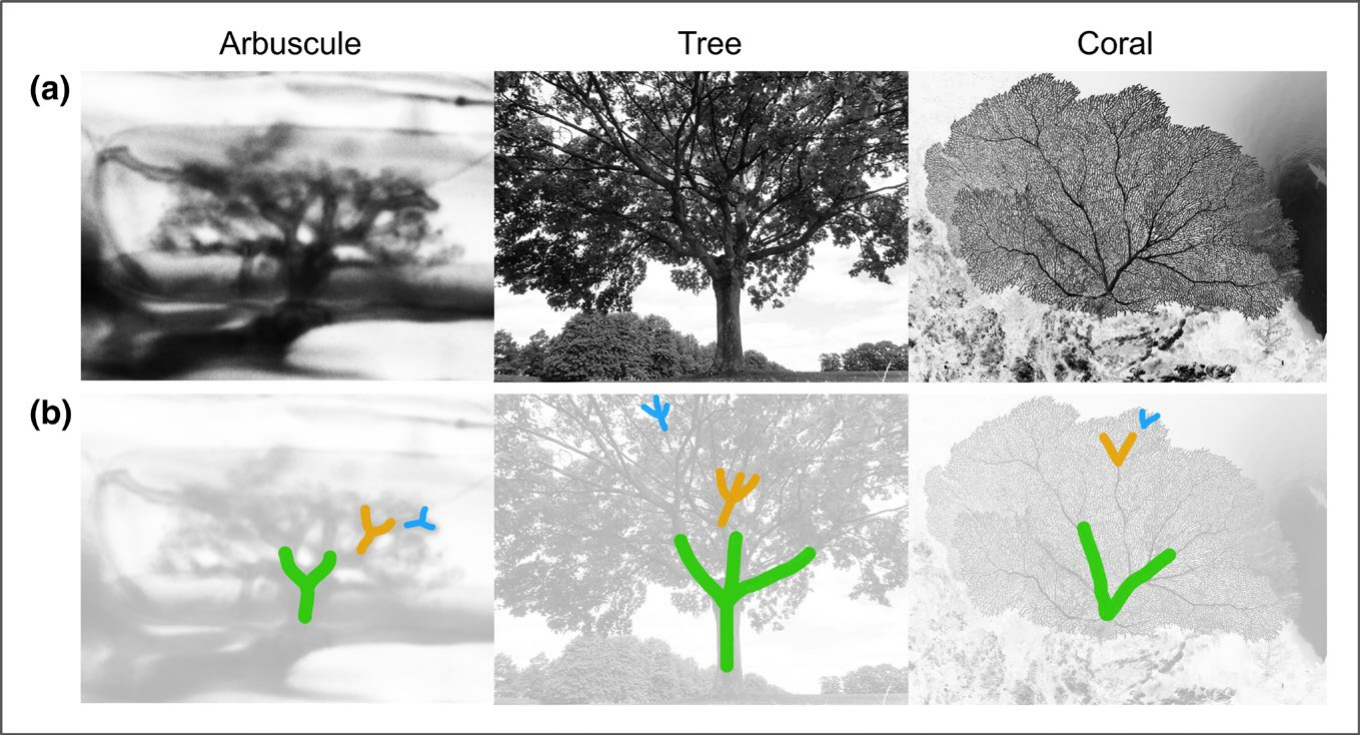
Branching fractals in nature. (a) Grayscale images of an arbuscule, (literally meaning “little tree”), a sycamore tree, and a sea‐fan coral. (b) Self‐similar patterns across different scales annotated on each image in different colours to show fractal‐properties. Coral photo courtesy of Warren Baverstock/Coral Reef Image Bank

One step further down the creative route is visualising the unseen in a more artistic fashion. Images of invisible organisms can seemingly hold as much natural beauty and inspiration as more classically “beautiful” macroscopic species (Figure 3c). Research into what makes both nature and art beautiful, appealing and engaging to humans supports this, highlighting the key role of fractals. These are patterns with self‐similar elements across different scales, such that the large‐scale pattern (e.g. a fern frond) is made up of smaller similar patterns (e.g. the leaves), which are in turn made up of even smaller similar patterns (e.g. the leaflets) and so on. It has been shown that the fractal dimensions (the complexity of the pattern) found frequently in nature and art correlate with the fractal dimension deemed preferable by people, as measured by physiological parameters, thought to be due to the evolution of the human visual system to make sense of natural surroundings (Spehar & Taylor, 2013). Just like attractive trees, pleasing plumage and stunning shells, overlooked and invisible organisms commonly feature fractal patterns. Examples include bacterial colonies (Rudge et al., 2013), filamentous fungi (Obert et al., 1990), plant roots (Dannowski & Block, 2005), and interestingly for our laboratory, arbuscules (Figure 4).

Harnessing the aesthetic beauty and inspiration of overlooked organisms in the form of images and art can therefore effectively attract and engage people. We have observed this first hand, where people outside of the mycorrhizal field have recognised the inherent aesthetics of arbuscules, frequently pointing out the similarity of arbuscules to trees, corals, and other more commonly‐considered beautiful natural structures (Figure 4). Images of overlooked organisms also hold the potential to inspire works of art (Nai & Meyer, 2016), which in turn have the power to engage and connect with a broader group of society, including those less attracted to more academic‐styles of outreach. While this can be facilitated by collaboration with artists (JIC, 2020), many researchers have gotten creative and produced beautiful and thought‐provoking works themselves (Harrower et al., 2018; Wolfson, 2020; Figure 3c).

### 3.4 Getting interactive

Lastly, visual products of research can be effectively shared with the public in an interactive manner. There are many options to get people involved in the visualisation process, for example by taking light microscopes to outreach events, under which people can explore stained mycorrhizal fungi inside cleared roots themselves. We also employ activities where people are tasked with sketching mycorrhizal networks between images of plants, and games, where players draw fungal hyphae through mazes and “build” plants to capture sunlight. Another way to get people involved in producing visuals of overlooked species is citizen science, for example the development of a “Mycorrhizal Home‐Kit” (Luginbuehl & Choi, 2017) involved people growing plants with and without AM fungi, noting down their observations, sending back the roots for fungal‐staining, and being able to view the resulting micrographs. Where direct contact with the public is not possible, “virtual microscope” programmes and softwares can be employed and online games created (e.g. Bonser et al., 2013; Coil et al., 2017; Lacey, 2017; Paulsen et al., 2010).

Letting people see otherwise invisible organisms for themselves acts as proof of existence (SeppÄNen & VÄLiverronen, 2003). Many invisible organisms lead such unbelievable lives that information shared by scientists can lead to disbelief (Bonfante & Desirò, 2017; Brouillette, 2020; “Microbiology by numbers”, 2011). The case of AM fungi exemplifies this. With no prior knowledge of what a fungus looks like, nor the structure of plant root, the concept of a fungus living *within* a plant root can be completely incomprehensible. We have found that showing people roots on a microscope slide, before letting them look at the resident fungi for themselves under the microscope holds the power to persuade even the most sceptical of the existence of AM symbiosis, and opens the gateway to engagement and appreciation.

Interactive visualisations can also facilitate learning, conjure a deeper level of engagement and are highly memorable (Lesen et al., 2016). What would you be more likely to remember—the time you were told that there are underground filamentous fungi connecting up the roots of plants, or the time you drew such a network, joining plants and foraging for nutrients in an illustrated soil? Sharing imagery via such hands‐on activities and games also links back to the importance of fun in the efficacy of raising awareness and understanding.

## 4 THE OUTCOMES: BENEFITS FOR PLANTS, PEOPLE AND PLANET

Whether via raw images, illustrations, artwork or viewing the organisms directly, we believe that visualisation of the invisible can bring broad benefits.

For plants, fungi and other overlooked organisms, visualising them for the public can increase the appreciation and compassion shown towards these often neglected species. The connection that results from viewing images and artistic representations of a fellow living species has been shown to increase the intrinsic value assigned to the subjects, as opposed to “use” or “commodity value” (Kalof et al., 2016), while the familiarity that comes with viewing an organism has been shown to positively affect conservation support for endangered species (Thomas‐Walters et al., 2020). Where children's interest and appreciation is captured by effective visualisations, a future of support for otherwise neglected organisms can be secured (Chawla, 2020; Cheng & Monroe, 2010).

For people, seeing invisible species can bring a wealth of benefits. Engaging with the natural world, even via images, has been shown to bring both emotional and psychological benefits including reduced stress, restored attentional capacity and increased happiness (Kaplan & Kaplan, 1989; Richardson & McEwan, 2018; Russell et al., 2013; Taylor et al., 2005). Equally, gaining a deeper understanding about the organisms around us, such as by visual means, has been identified as a pathway to wellbeing (Bragg et al., 2015). Images of invisible organisms can also trigger an interest in the species, area of research, or science more generally, potentially increasing the uptake of neglected subjects and modules in school and university, and bolstering under‐manned and under‐funded areas of research (Hawksworth, 2009; Stagg et al., 2009). Stimulating this interest can also increase scientific literacy in society (Timmis et al., 2019).

The benefits of visualising the invisible may extend to the level of the planet. Visually engaging with nature, including via images of the otherwise invisible, can strengthen people's feelings of connectedness‐to‐nature, which has been shown to motivate more environmentally‐friendly and compassionate behaviours (Alcock et al., 2020; Geng et al., 2015; Whitburn et al., 2020). Engaging with the beauty of nature, be it directly, via images, or through art, is proposed to stimulate thoughts about ones relationship with nature, impact on the environment and purpose of life (Kaplan & Kaplan, 1989; Passmore & Holder, 2017; Richardson & McEwan, 2018; Russell et al., 2013). Sharing images of overlooked organisms may therefore inspire greater consideration and care for the nature surrounding us.

## 5 CONCLUDING REMARKS

By highlighting the great potential of scientific visuals to increase awareness, interest and appreciation of unseen species, the ease and diversity of ways in which it can be achieved, and the broad and far‐reaching benefits that can result, we aim to have made a convincing case for more researchers to share their images beyond their fields of study. Through examples of our own outreach activities involving AM symbiosis, and those of other plant and microbial researchers, we hope to have provided some inspiration and starting points for those now interested in getting their visuals into the public sphere. While AM symbiosis has been used as an example throughout, the principles and ideas are applicable far more extensively, to any area of biology where the objects of study, from ecosystems to individuals to subcellular structures, are not naturally visible or commonly seen by the public. Equally, our focus has almost solely been on visual modes of communication, due to the fact that the raw material is already available in abundance and therefore presents an untapped resource; however we recognise that a holistic approach (e.g. including written text, audio, touch and smell) can bring further benefits to engaging people with the invisible.

So let's not keep our insights into the invisible fractions of the natural world to ourselves, but instead seize the exciting and important opportunity to show‐off the otherwise unseen for the benefit of plants (and other overlooked organisms), people and planet.

## AUTHOR CONTRIBUTIONS

J.M wrote the manuscript. U.P edited the manuscript.
